# Accuracy Improvement of Automatic Smoky Diesel Vehicle Detection Using YOLO Model, Matching, and Refinement

**DOI:** 10.3390/s24030771

**Published:** 2024-01-24

**Authors:** Yaojung Shiao, Tan-Linh Huynh, Jie Ruei Hu

**Affiliations:** 1 Department of Vehicle Engineering, National Taipei University of Technology, Taipei 10608, Taiwan; 2 Railway Vehicle Research Center, National Taipei University of Technology, Taipei 10608, Taiwan

**Keywords:** deep learning, license plate, smoke detection, smoky diesel vehicle, YOLO

## Abstract

The detection of smoky diesel vehicles is a key step in reducing air pollution from transportation. We propose a new method for identifying smoky vehicles that proceeds in three stages: (1) the detection of vehicle shapes, license plates, and smoke regions; (2) the implementation of the two matching techniques based on the smoke region–vehicle shape and smoke region–license plate relationships; and (3) the refinement of the smoke region detected. The first stage involves the evaluation of various You Only Look Once (YOLO) models to identify the best-fit model for object detection. YOLOv5s was the most effective, particularly for the smoke region prediction, achieving a precision of 91.4% and a mean average precision at 0.5 (mAP@0.5) of 91%. It also had the highest mean mAP@0.5 of 93.9% across all three classes. The application of the two matching techniques significantly reduced the rate of false negatives and enhanced the rate of true positives for the smoky diesel vehicles through the detection of their license plates. Moreover, a refinement process based on image processing theory was implemented, effectively eliminating incorrect smoke region predictions caused by vehicle shadows. As a result, our method achieved a detection rate of 97.45% and a precision of 93.50%, which are higher than that of the two existing popular methods, and produced an acceptable false alarm rate of 5.44%. Particularly, the proposed method substantially reduced the processing time to as low as 85 ms per image, compared to 140.3 and 182.6 ms per image in the two reference studies. In conclusion, the proposed method showed remarkable improvements in the accuracy, robustness, and feasibility of smoky diesel vehicle detection. Therefore, it offers potential to be applied in real-world situations.

## 1. Introduction

Air pollution is a serious public health concern [1] that largely stems from gas emissions from diesel-powered vehicles and heavy machinery. Thus, reducing pollution from diesel vehicles is essential for improving air quality [2]. Initial efforts to mitigate this problem involved two conventional methods for detecting smoky diesel vehicles: human inspection (either directly on roads or through surveillance cameras by environmental protection staff or traffic police) and routine exhaust gas analysis at government vehicle testing centers. Despite the simplicity of these methods, they are labor-intensive and inefficient, particularly in situations with dense, fast-moving traffic. In addition, periodic examination using an exhaust gas analyzer, although effective, requires costly equipment that could interfere with vehicle operations [3,4].

Advancements in image processing and deep learning have led to the application of computer vision techniques for the automatic detection of smoky vehicles. The first approach focuses on image processing. Pyykonen et al. proposed a multi-camera system comprising a far infrared camera to locate the vehicle’s exhaust pipe and a high-resolution visible-wavelength camera for exhaust area analysis [5,6]. Despite its utility, this system has several weaknesses, including high costs, poor waterproofing, limited durability, and daytime color distortion. The second approach involves convolutional neural network (CNN) algorithms. Kundu et al. proposed a framework utilizing three CNN models: Inception-V3, MobileNet-V2, and InceptionResNet-V2, to classify smoky vehicles [7]. This method employs a consensus mechanism where a “smoky vehicle” classification is confirmed if at least two out of the three models agree with this assessment. This consensus mechanism offers robustness but at a considerable increase in computational cost and reduction in sensitivity. To address these limitations, Kundu et al. proposed a new method involving smoke synthesis for training data augmentation and the integration of a lambda-implemented attention-based detection network into the You Only Look Once (YOLO) version 5 algorithm [8]. However, this method still struggles with high false alarm rates, likely due to the synthesized smoke’s inability to accurately represent real-world variations in smoke density and shape. The third approach involves a combination of image processing and CNN algorithms, as exemplified in the works of Tao et al. [9,10,11,12,13]. This dual-stage method first identifies key regions on vehicles for smoke detection and then classifies vehicles into “smoke” and “nonsmoke” classes. The approach incorporates various image processing methods, such as local binary patterns, histograms of oriented gradients, integral projections, and motion boundary histograms. In addition, Wang et al. [14] and Yuan et al. [15] proposed a two-stage CNN process, using YOLOv3 for key region detection and either a multi-region convolutional tower network for fine-grained classification [14] or the vision transformer (ViT) [16] for smoky vehicle identification [15]. These approaches are feasible and robust but are limited by long computation times and low sensitivity for smoke outside the predetermined key regions (e.g., at the right or left side or on the exhaust pipe of the vehicle). Moreover, Peng et al. [17] further refined smoky vehicle detection using a three-stage procedure involving CNN models for smoke region and vehicle shape detection, a matching algorithm to reduce false positives, and a short-term spatial–temporal network for final verification. This method effectively addresses the problem with shadows but requires considerable processing time due to the use of three CNN models.

Furthermore, the YOLO framework has undergone substantial advancements over the 2010s, with numerous versions released [18,19,20,21]. These iterations have demonstrated exceptional effectiveness in recognizing a variety of objects [22,23,24]. Studies employing various YOLO versions or YOLO-based models specifically for the detection of vehicle smoke or smoky vehicles [8,14,15,25] have demonstrated the robustness, stability, accuracy, and speed of YOLOv5. Therefore, we chose YOLOv5 in our study.

To overcome the aforementioned issues, in this paper, we propose an automatic method for detecting smoky diesel vehicles that potentially reduce the processing time, which is essential for real-time application and the maintainence of high performance. First, we collected a high-resolution large-scale real-world dataset of 6815 smoky vehicle images and 24,930 corresponding annotations, which enhances the quality of the training dataset. Second, we determined a single version of YOLOv5 that was the most effective in detecting simultaneously three objects, including vehicle shapes, license plates, and smoke regions, which substantially save the processing time, compared to using YOLO versions simultaneously for detecting those objects, as proposed in previous studies [17]. Third, we proposed two pair-wise matching algorithms, namely smoke–vehicle and smoke–license plate, which can increase the prediction accuracy, compared to previous studies that did not apply matching or used one matching algorithm, and it does not cause computational burden [9,12,17]. This approach can decrease the false positive rate (i.e., eliminating the detected wrong smoke regions) and also reduce the false negative prediction of diesel vehicle missed cases by additionally using the latter matching algorithm. Moreover, matching also reduces the confusion induced by irrelevant objects in each frame. Finally, we proposed a refinement method to exclude the incorrect detection of smoke objects due to a vehicle’s shadow. This simple technique can improve the accuracy with a remarkably lower time cost, compared to CNN algorithms, which have been applied in prior research.

## 2. Automatic Smoky Vehicle Detection Method

**Figure 1:**
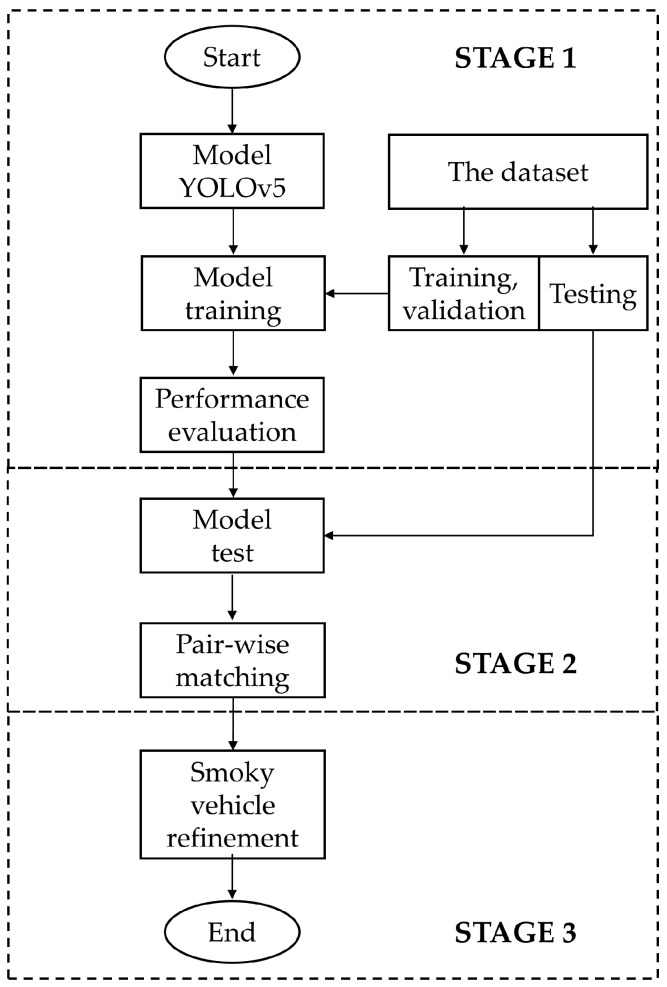
Flowchart of the proposed method of automatic smoky vehicle detection.

Our automated smoky diesel vehicle detection system proceeds in three stages (Figure 1). The initial stage involves data collection, labeling (annotation), and the selection of the most appropriate YOLOv5 version for detecting smoke regions, license plates, and vehicle shapes. The second stage involves matching smoke with the vehicle shape and the license plate. The final stage centers on refining the detection of smoky vehicles.

### 2.1. Smoky Diesel Vehicle Datasets

High-quality data are required to effectively train CNN models, but data on vehicle smoke are scarce. Although large public datasets exist for wildfire smoke, such as KMU Fire-Smoke [26], Mivia Fire-Smoke [27], and VSD [28], wildfire smoke differs markedly from vehicle smoke in terms of characteristics such as density, shape, direction, and location. Moreover, the forest background in these datasets does not correspond well to complex urban traffic environments [17], potentially leading to a high alarm rate (approximately 13%) when such data are used for vehicle smoke detection [12]. On the other hand, existing traffic surveillance systems offer a valuable source of data. Conventionally, cameras are set up at a high position, e.g., on the top of traffic lights. This offers several advantages, including a broader view of traffic vehicles, the use of available traffic facilities, and reducing obscurity due to heavy traffic. However, this placement often produces images with large shadow areas behind vehicles, making smoke detection difficult. To overcome this challenge, we proposed to set up high-resolution cameras at lower vantage points. This strategic placement—inside environmental protection cars, at roadside stations, or on traffic light poles—significantly improved the detection of smoke by reducing shadow areas, minimizing reflections from wet roads, and enhancing the sharpness of smoke regions.

**Figure 2:**
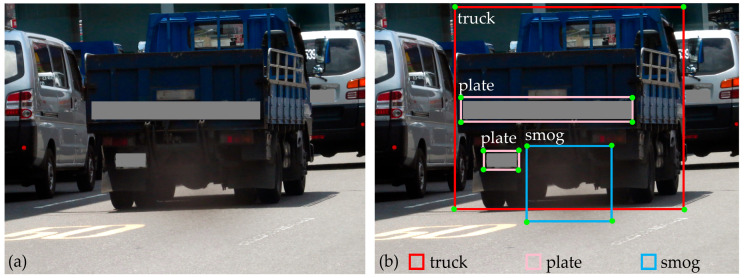
A typical example of (**a**) an input image and (**b**) its labels.

To address the limitations of existing datasets and improve the data’s diversity and richness, the cameras were set up and collected data on several types of roads in several cities in Taiwan. Data were collected over different periods of daytime (from dawn to dusk) for several months. We selected the most suitable images that fit our research objective to form the final dataset consisting of 6815 images of smoky diesel vehicles. They are high-resolution images (180 dots per inch) with a size of 2816 × 2112 pixels. The dataset captured various types of diesel vehicles (e.g., light truck, medium truck, heavy truck, specialized diesel vehicle, and bus) with a diverse background of roads (e.g., suburban, urban, and highway). In addition, the dataset was enriched with various features of smoke (e.g., shape, color, and density). The data collected cover different good weather conditions, including sunny, clear, partly cloudy, or overcast days; however, we did not collect data in bad weather, such as rain and fog; we have acknowledged this limitation and proposed future research to address this issue. A typical image of a smoky diesel vehicle captured on the roads is illustrated in Figure 2a. The LabelMe toolbox (https://github.com/CSAILVision/LabelMeAnnotationTool, accessed on 30 March 2023) was used to annotate the data into three classes: “truck” (delineated by the chassis and tires of the diesel vehicle), “plate” (the diesel vehicle’s license plate), and “smog” (the diesel vehicle’s smoke region). Given the predominance of trucks in the dataset, along with the presence of several buses and a few sport utility vehicles, we labeled the class of diesel vehicle objects as “truck”. An example of these annotated objects is presented in Figure 2b.

The final dataset consisted of 6815 images of smoky diesel vehicles with approximately 24,930 corresponding annotations. These were then randomly divided into training and validation sets, maintaining an 80:20 ratio (Table 1).

### 2.2. YOLOv5 Models Analysis and Evaluation

#### 2.2.1. YOLOv5 Architecture

**Figure 3:**
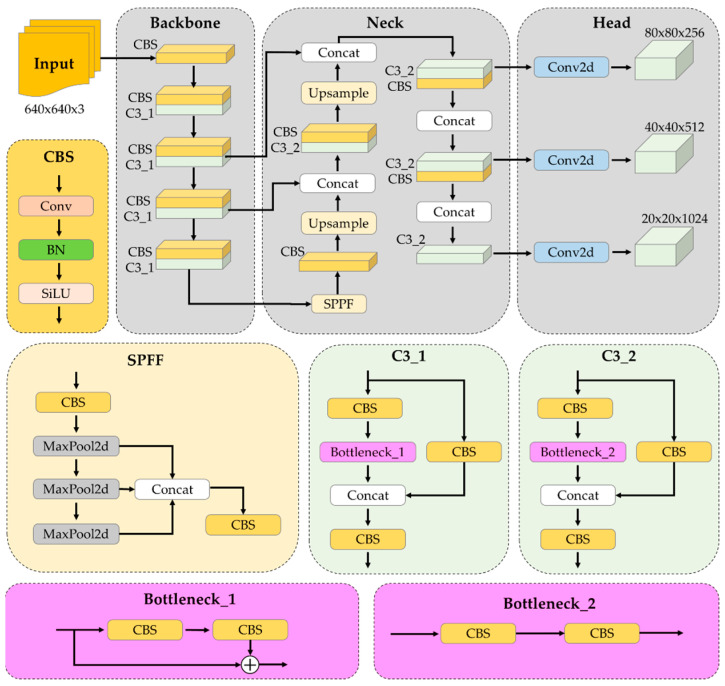
Network architecture of YOLOv5.

YOLOv5 has five variants: YOLOv5n, YOLOv5s, YOLOv5m, YOLOv5l, and YOLOv5x [23]. These variants are designed following the composite scaling model, inspired by EfficientDet [29]. These YOLOv5 variants primarily differ in their depths and widths, which are determined by depth-multiplier and width-multiplier settings in the YAML configuration file. YOLOv5′s architecture has four essential components (Figure 3) [23]:

1. Input augmentation: This component focuses on diversifying the input data. Techniques used include Mosaic; Copy and Paste; random affine transformations (such as Rotate, Scale, Pan, Horizontal Flip, and Cut); MixUp; and adjustments to the Hue, Saturation, and Value channels.
2. Backbone: The backbone is responsible for feature extraction. It processes the input image through a sequence of 6 × 6 Conv2d layers. The innovative CSPDarknet53 architecture enhances the learning capability and reduces the computational cost of the CNNs [30].
3. Neck: This component is responsible for multiscale feature fusion within the feature maps through the integration of the spatial pyramid pooling fast (SPPF) layer and the new cross-stage partial-path aggregation network (CSP-PAN) [31]. SPPF effectively integrates features of varying scales into a fixed-sized feature map, thereby accelerating network computations. To process features at different scales, the convolution-BatchNorm-SiLu and C3 layers are employed, incorporating batch normalization and SiLu activation functions.
4. Head: The YOLOv5 Head component, resembling the YOLOv3 Head, handles anchor-based predictions, object classification, and bounding box regression. Anchors are utilized to predict bounding boxes; classification is employed to categorize objects in these boxes; and regression is employed to determine the precise locations and dimensions of the bounding boxes. Overlapping bounding boxes are minimized using non-maximum suppression (NMS).

#### 2.2.2. Evaluation Metrics in YOLOv5

**Figure 4:**
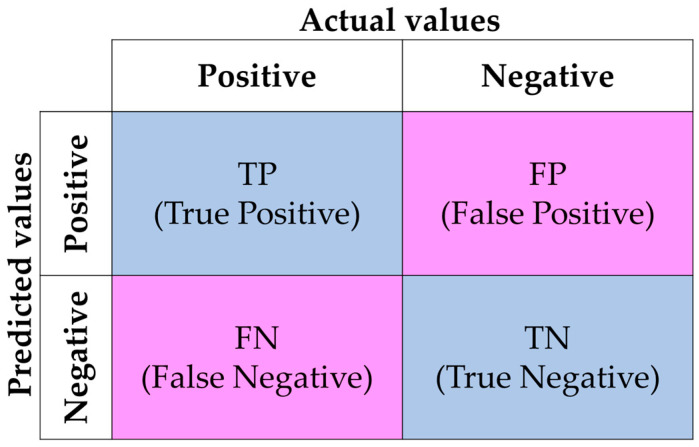
The confusion matrix of true positive, false positive, true negative, and false negative.

YOLOv5 utilizes a comprehensive set of evaluation metrics to assess its performance in accurately classifying and locating objects within images. These metrics include precision, recall (or detection rate (DR)), and false alarm rate (FAR). *Precision* reflects the accuracy of the positive predictions made by the model. It represents the ratio of correctly predicted objects to all predicted objects, as illustrated in Equation (1). *Recall* (or *DR*), also known as the true positive rate or the missing positive prediction rate, is defined as the proportion of true positive predictions out of the total actual objects, as specified in Equation (2). The *FAR* is used to evaluate the model’s ability to classify objects that specifically pertain to smoky vehicles. The *FAR*, in the context of the present study, indicates the probability that a vehicle is incorrectly classified as a smoky one, as specified in Equation (3). In this study, a true positive (*TP*) is a correct identification of the objects in the image, a false positive (*FP*) is an incorrect identification of the objects in the images, a true negative (*TN*) is a correct identification of the objects that are not in the image, and a false negative (*FN*) is an incorrect identification of the objects that are not in the image. (*TP* + *FP*) is the total number of predicted objects in the images, (*TP* + *FN*) is the total number of objects that are actually in the images, and (*FP* + *TN*) is the total number of objects that are indeed not in the images. The relationships between *TP*, *FP*, *TN*, and *FN* are illustrated in Figure 4.

(1) $$Precision=\frac{TP}{TP+FP}$$

(2) $$Recall=DR=\frac{TP}{TP+FN}$$

(3) $$FAR=\frac{FP}{FP+TN}$$

Moreover, the average precision (*AP*) is the area under the precision–recall curve, and is used to evaluate the average accuracy of a single category. The *AP* is defined by Equation (4), where *K* is a given set of thresholds and $R_{i}$ and $P_{i}$ are the recall and precision at each threshold *i*, respectively. The mean AP (*mAP*) represents the mean value of the *AP* across all classes, ranging between 0 and 1, as outlined in Equation (5).

(4) $$AP=\sum_{i=1}^{K}\left(R_{i}-R_{i-1}\right)P_{i}$$

(5) $$mAP=\frac{\sum AP}{Num(class)}$$

#### 2.2.3. Experiment Environment

The models were trained on a system running the Windows 11 Professional 22H2 x64 operating system with a Nvidia RTX 3080 graphics card (Table 2).

### 2.3. Matching Techniques

**Figure 5:**
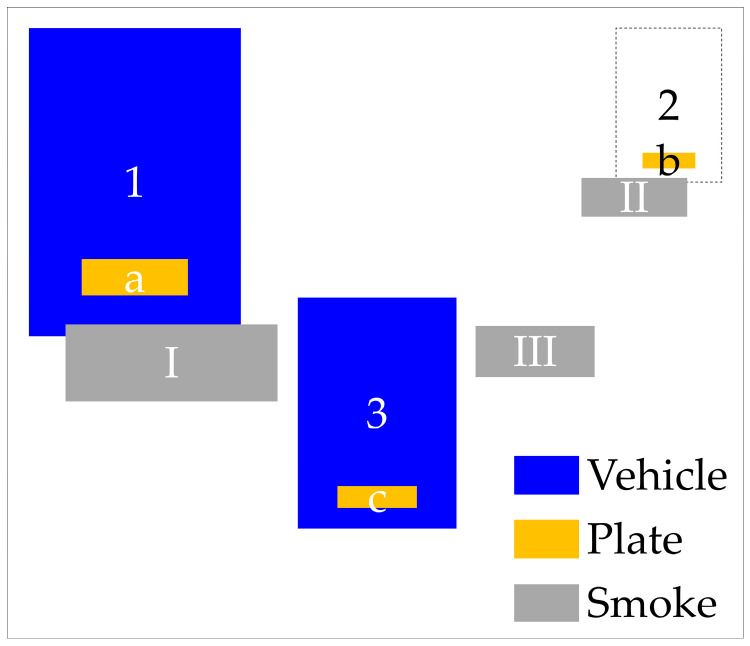
A typical example for pair-wise matching techniques.

The prediction of smoky vehicles that is solely based the smoke region seems insufficient. The matching techniques have been introduced to reduce FPs in smoke predictions, for instance, the ‘smog–truck’ matching techniques in Peng et al. [17]. However, this approach might miss out the case that the truck is not detected. Therefore, we propose two pair-wise matching techniques: one for the smoke region–license plate (referred to as “smog–plate”) relationship, and another for the smoke region–vehicle shape (referred to as “smog–truck”) relationship. To the best of our knowledge, this is the first study that introduces the vehicle’s license plate as a matching object to smoke regions. The second matching technique has the aim of reducing FNs in vehicle shape predictions. The underlying principle of the two matching techniques is that each smoke zone originates from a specific vehicle, which can be identified either by its shape, its license plate, or both. These matching algorithms are summarized in Table 3 and illustrated in Figure 5. In our analysis, we identified 13 potential cases, outlined in Table 3, which included 5 and 8 cases where the models identified the object as a smoky and nonsmoky vehicle, respectively. These cases were established by comparing the vehicle regions, license plate regions, and smoke regions in each frame. For instance, in the eighth case, the model may fail to detect a smoky vehicle if it only relies on the vehicle’s shape to do so; this problem is mitigated by incorporating the license plate in the identification process. Similarly, the thirteenth case demonstrates a situation where the smoke and vehicle shape are detected, but the smoke originates from a different vehicle, identifiable through its license plate. As a result, the no-matching approach might lead to the incorrect prediction of the smoky vehicle (FPs), as in cases 4, 7, 9, and 10. Using the single-matching method (smog–truck) might result in the incorrect prediction of the smoky vehicle (FNs), as in cases 8 and 13. Using the two proposed pair-wise matching techniques can prevent incorrect detection in such cases.

An illustrative example of these matching techniques is presented in Figure 5. This example features two smoke regions emitted from vehicles (labeled as I and II), three vehicles (labeled as 1, 2, and 3), three license plates (labeled as a, b, and c), and one incorrectly predicted smoke region (labeled as III). Specifically, the model failed to detect the geometry of the vehicle (2) and mistakenly identified region III as a vehicle-emitted smoke region. Through our matching techniques, the system correctly identified two smoky vehicles: a vehicle shape–license plate–smoke region (1–a–I) and license plate–smoke region (b–II). It also correctly disregarded region III and correctly categorized vehicle 3 as a nonsmoky vehicle. Thus, our matching method effectively reduced the occurrence of FNs and FPs.

The real vehicle is identified through the vehicle region, the license plate region, or a combination of both, thus improving the accuracy of actual vehicle detection. The intersection over union (IoU), distance thresholds, and bounding box coordinates are used to analyze the relative positions in three comparisons: license plate–vehicle shape, smoke–vehicle shape, and smoke–license plate. In cases where the IoU is applied to the license plate–vehicle shape relationship, a nonzero IoU indicates the license plate is within the vehicle region, implying they belong to the same vehicle. Conversely, an IoU of 0 suggests they are from two distinct vehicles. In addition, distance thresholds ($D_{s-v},D_{s-p}$) and bounding box coordinates are used to determine the relationship of smoke with the vehicle shape and the license plate. Because vehicle shapes and smoke regions differ in size, specific distance thresholds are set to ascertain whether the smoke is emitted from a particular vehicle. This is estimated by the distance between the bottom-middle points of the vehicle region and the top-middle points of the smoke region. Likewise, the relationship between the smoke and the license plate is established based on the distance between the bottom-middle points of the license plate region and the top-middle points of the smoke region.

### 2.4. Smoke Region Refinement

**Figure 6:**
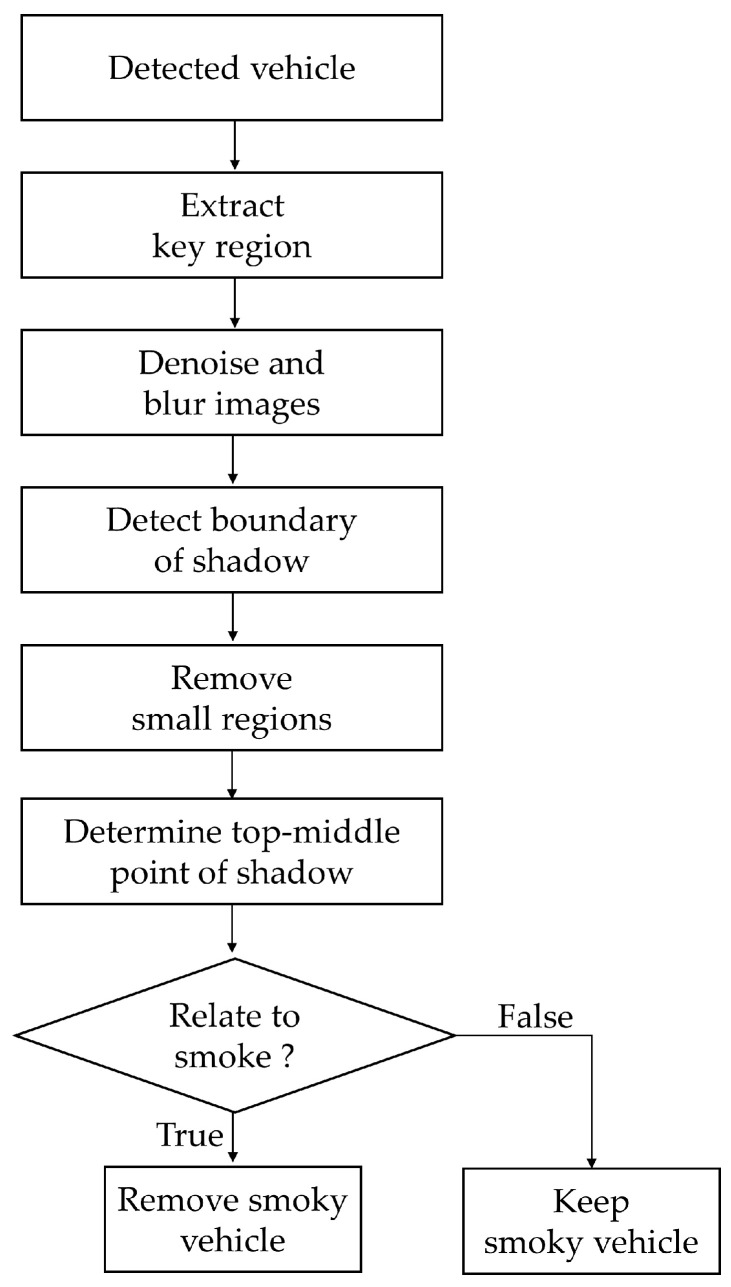
The procedure of smoke region refinement.

**Figure 7:**
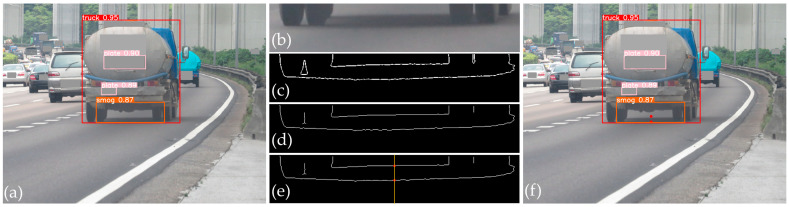
The procedure of smoke refinement: (**a**) detecting a smoky vehicle, (**b**) extracting a blurred key region, (**c**) determining the shadow’s border using the Canny and Otsu algorithms, (**d**) applying the skeleton model, (**e**) marking the middle points of the shadow’s border, and (**f**) comparing the locations of the smoke and shadow regions.

To further minimize the FPs and FNs, particularly those caused by shadows being misidentified as vehicle smoke regions, we proposed a technique for smoke refinement. This process includes several steps to distinguish between actual smoke and shadow artifacts. First, vehicle recording cameras were strategically placed at low angles to enhance vehicle ground clearance visibility and thereby reduce shadow areas. Second, image processing principles were applied to images with matched objects to identify the border of the shadow and assess its relation to the detected smoke region. This section presented a flow chart of the smoke region refinement procedure (Figure 6) as well as an example with a detailed explanation to illustrate this procedure (Figure 7).

Extract key region: After the smoky vehicle was identified with both the smoke region and the shape of the vehicle (Figure 7a), the key region had the same width as the detected vehicle and a height of up to 200 pixels centered around the bottom line of the vehicle’s rectangle. These settings were designed to encompass all potential instances where shadows are present. The key region in this step might contain one or more objects as follows: the shadow, the bottom part of the vehicle, a part of the road background, and the smoke region.

Denoise and blur images: To enhance the visualibility of the shadow region, we need to eliminate the noise caused by the smoke region and road background from the key region. For this purpose, a Gaussian filter method was applied to denoise, blur, and smoothen the key region. As a result, this step considerably reduces the appearance of small edges, points, and tiny areas formed by the road or smoke region (Figure 7b).

Detect shadow’s boundary (i.e., border of shadow): The key region at this stage often contained two gray levels: black (e.g., the shadow and wheels) and gray (e.g., the road); thus, the Otsu’s threshold was applied to convert the key region into a black and white image: the background in black, containing the shadow, vehicle wheels, or other black details; the foreground in white, consisting of the ground clearance, road, or other details in bright colors. The Otsu method provided a threshold as an output in an automatic and adaptive manner, based on the gray histogram [32]. The Canny was then applied to detect the shadow’s border and the remaining elements in the key region. This method is commonly applied in real-world settings due to its ability to accurately determine both the unilateral response and edge location [33] (Figure 7c).

Refine shadow’s boundary: The key region might contain undesirable tiny areas or short edges, and thick shadow edges. Therefore, the skeleton method [34] was used to thin out the shadow border obtained from the previous step to enhance the accuracy in locating the shadow border. In addition, undesirable tiny areas were also removed based on the contour area (Figure 7d).

Determine top-middle point of shadow: With cameras sett up at low positions, the upper and lower borders of shadow were relatively parralel. Using this feature, we proximated the location of the upper and lower shadow boundaries by the coordinates of the top- and bottom-middle points of the shadow. These two points were the intersections between the shadow’s border and the center vertical line (orange line) of the key region (Figure 7e). The top-middle point was used to assess the relation with the smoke region in the next step.

Relate to smoke?: This step is completed to assess the relationship between the smoke and shadow regions by comparing the relative locations of the smoke and shadow regions. For this purpose, we first determined the coordinates of the top-middle point of the smoke region obtained by YOLOv5 in stage 1 (Figure 7f). Given that the vehicle’s exhaust pipe is higher than its shadow due to ground clearance, the top-middle point of the smoke region is expected to be higher than that of the shadow region. Let *D_t_* be the distance between the top-middle points of the shadow and smoke regions, with *T*_t_ representing the threshold for *D_t_*. A *D_t_* value less than T_t_ indicates that the smoke is a shadow, whereas a value equal to or greater than *T*_t_ indicates vehicle-emitted smoke. For this study, we set *T_t_* to 10 pixels, accounting for tolerances at the shadow edge and smoke region detection.

## 3. Results and Discussion

### 3.1. Performance of YOLOv5 Models

**Figure 8:**
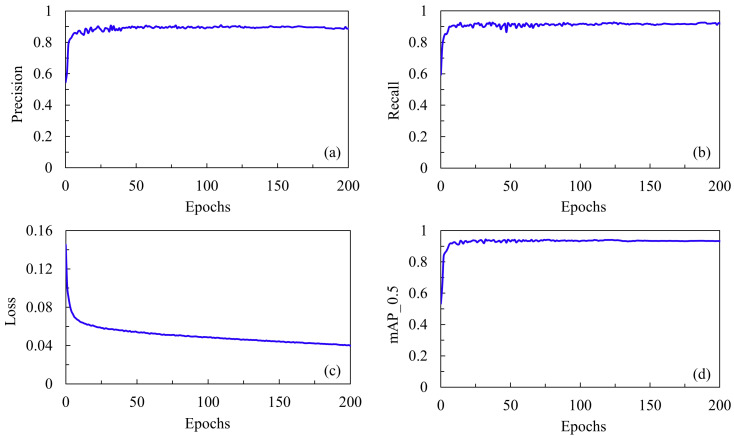
The mean values of four training performance metrics of YOLOv5s model for all three classes: (**a**) precision, (**b**) recall, (**c**) loss function, and (**d**) mAP@0.5.

**Figure 9:**
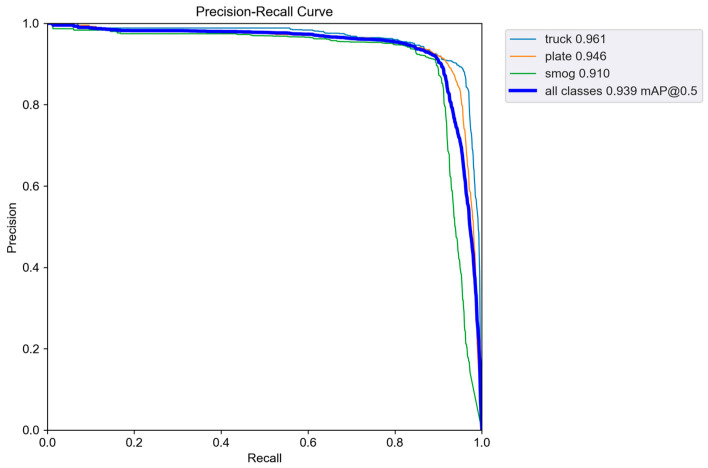
Relationship between precision and recall of three classes with YOLOv5s model.

We evaluated the performance of four YOLOv5 models in detecting the three classes (“truck”, “plate”, ”smog”) and used the mean values across these classes, given their equal importance in our study’s context. Table 4 outlines the training performance metrics for the four YOLOv5 models. The YOLOv5s model had the best precision and mAP at a threshold of 0.5. Conversely, the YOLOv5m model excelled in recall and mAP across a range from 0.5 to 0.95. Regarding the detection of “smog”, the central object of interest, the YOLOv5s model again exhibited the highest performance in all metrics (91.4%, 88.3%, 91%, and 42.1% for precision, recall, mAP at 0.5, and mAP from 0.5 to 0.95, respectively). The overall training performance of the YOLOv5s model is depicted in Figure 8 and Figure 9. This model demonstrated stability and high performance within 200 epochs. As illustrated in Figure 8, the mean values of precision, recall, and mAP@0.5 for “truck”, “plate”, and “smog” were all above 0.90, whereas the loss function gradually decreased to below 0.04. Furthermore, Figure 9 illustrates the relationship between precision, recall, and mAP@0.5 for each class when the YOLOv5s model was used. Notably, the recall for “smog” was the lowest among the three classes.

### 3.2. Evaluation of the Proposed Method

**Figure 10:**
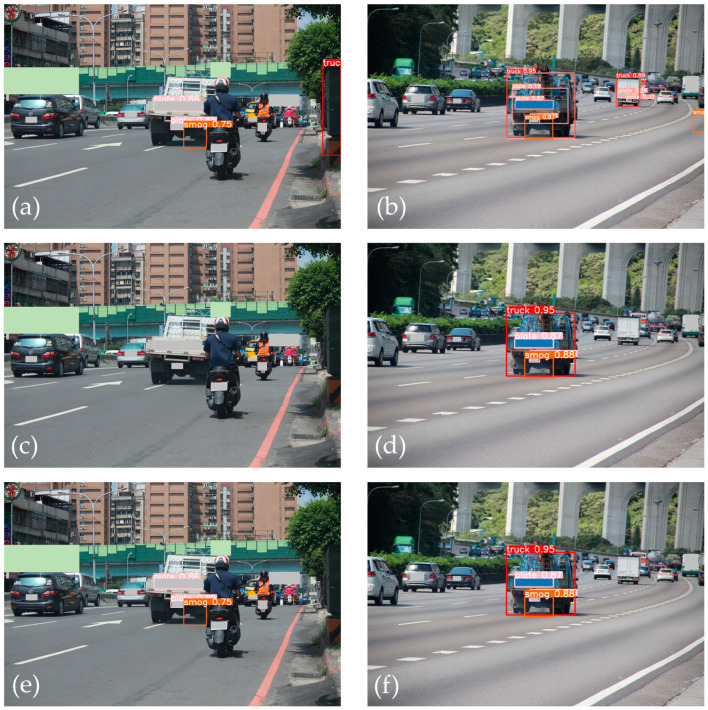
Examples of prediction results: (**a**,**b**) using YOLOv5s; (**c**,**d**) using YOLOv5s combined with smoke–vehicle method; (**e**,**f**) using YOLOv5s combined with the two matching techniques.

**Figure 11:**
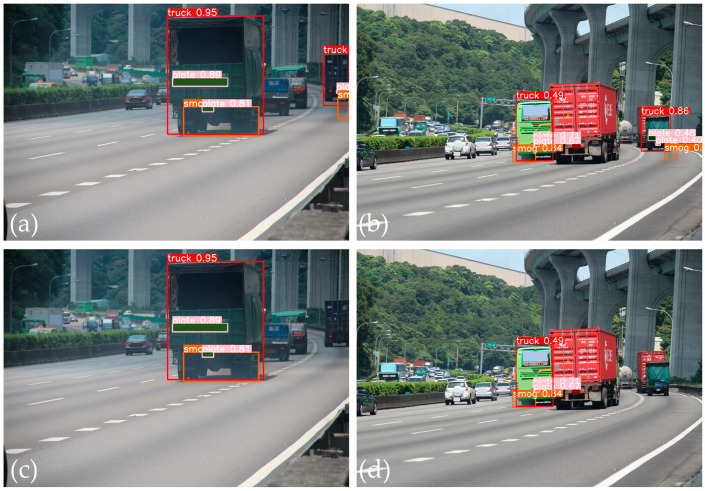
Examples of prediction results: (**a**,**b**) using the combination of the YOLOv5s and the two matching techniques; (**c**,**d**) using the combination of the YOLOv5s, the two matching techniques, and the smoke refinement.

The effectiveness of our proposed method for smoky vehicle detection was evaluated through a series of tests, the results of which are illustrated in Figure 10 and Figure 11. Figure 10 presents the prediction results for two examples of smoky vehicle detection using different detection methods. Using only YOLOv5s, the model incorrectly detected the vehicle shape and missed the shape of a smoky vehicle (Figure 10a). As depicted in Figure 10b, in this instance, this method incorrectly detected the smoke region. When combined with the single smoke–vehicle matching technique, the model improved by eliminating incorrect vehicle shape detection, but still missed a smoky vehicle (Figure 10c). However, as illustrated in Figure 10d, this method successfully detected a smoky vehicle in another instance. The integration of YOLOv5s with two pair-wise matching techniques (Figure 10e,f) resolved these detection problems more effectively, correctly identifying smoke regions and eliminating nonsmoky vehicles. Figure 11 further demonstrates the refinement of this approach. Even with the combination of YOLOv5s and two matching techniques, some incorrect smoke region detections occurred due to road background noise or shadows near the detected vehicle (Figure 11a,b). This problem was addressed by the addition of smoke region refinement (Figure 11c,d). 

For a more comprehensive evaluation, 164 images containing 265 diesel vehicles (147 nonsmoky vehicles and 118 smoky vehicles) were selected for testing. The test results, measured in terms of the *TP*, *FP*, *TN*, and *FN* predictions for smoky and nonsmoky vehicles, where the *TP* is the correct positive prediction for the smoky vehicle, the *FP* is the incorrect positive prediction for the smoky vehicle, the *TN* is the correct negative prediction for the nonsmoky vehicle, and the *FN* is the incorrect negative prediction for the nonsmoky vehicle, are summarized in Table 5. This table presents the precision, the FAR, and processing time results of the different methods on this testing dataset. Our proposed method, which is based on YOLOv5s with the “smog–plate” and “smog–truck” matching techniques, and smoke region refinement, demonstrated superior precision and FAR compared with the use of YOLOv5s with only a single smoke–vehicle matching technique or each of the two matching techniques used independently. Specifically, our method achieved a higher DR (10.76%), precision (12.99%), and a lower FAR (10.21%) than YOLOv5s combined with just the smoke–vehicle shape matching. It also outperformed the combination of YOLOv5s with two matching techniques, with a higher precision (2.23%) and a lower FAR (2.04%). Regarding the computational efficiency, our method maintains a rapid average inference time for each frame, even when the two matching and refinement steps have been added. Specifically, it takes 85 ms to process an image, while the processing time is 53 ms per image for the approach of YOLOv5 combined with the smoke–vehicle matching, and 55 ms per image for YOLOv5 combined with the two matching methods.

### 3.3. Comparison to the State of the Art

To provide a more comprehensive view, we compare the proposed method with two other popular methods proposed by Tao et al. [12] and Peng et al. [17] (Table 6). Among several studies on smoke detection, these two references studied “smoky vehicles”—the same object as in the present study and using similar approaches, involving the combination of CNN algorithms and image processing. All measurements were performed using CPU: AMD Ryzen 9 5950X 16-cores 3.40 GHz CPU for the present study, Intel Xeon E5-2678 v3 12-cores 3.1 GHz CPU for Peng et al. [17], and Intel(R) Core(TM) 2 Duo2.4 GHz i7 CPU for Tao et al. [12]. Note that different hardware might lead to differences in the processing time; however, the reduction in the processing time is also determined by the methodology. Particularly, instead of using CNN models to reduce the false prediction, we used an additional matching technique (smoke–plate) and a refinement stage, which consume a small amount of time (Table 5).

Nowadays, a camera that is often used in surveillance can capture approximately 24 frames per second, equivalent to a processing time of 42 ms per frame (https://web.archive.org/web/20110708155615/http://www.cinemaweb.com/silentfilm/bookshelf/18_kb_2.htm, accessed on 10 October 2023). In order to apply to a real-time problem, a processing time close to this number is desirable. Our proposed method can process an input frame within 85 ms, which is substantially faster than that of the two previous methods. In addition, our approach achieves a higher DR and precision by 8.81% and 7.2%, compared to [12], respectively, and by 20.32% and 14.14%, compared to [17]. In addition, our method achieves a lower acceptable FAR, compared to [12]. A direct comparison of our results with the others might be difficult due to differences in the test data and computed capacities between these studies. Nevertheless, this provides an overview of our results in relation with those of existing methods, suggesting that the proposed method has potential to be applied in real-world situations.

## 4. Conclusions

In this paper, we implemented a new method for identifying smoky vehicles, including three stages: (1) detecting the vehicle shape, license plate of the vehicle, and smoke region; (2) matching smoke region–vehicle shape, and smoke region–license plate; and (3) refining the smoke regions. This paper suggested that the YOLOv5s version was the most suitable version for detecting those objects. The effectiveness of two matching techniques was confirmed through the reduction in false negatives for the smoky vehicles and the improvement of the true positive predictions for the smoky diesel vehicles by detecting their license plates. Moreover, the refinement for the smoke region using image processing theory shows usefulness in eliminating the incorrect prediction of the smoke region due to vehicle shadow. As a result, our method achieved a detection rate of 97.45% and a precision of 93.50%, which are higher than that of the two existing popular methods, and produced an acceptable false alarm rate of 5.44%. Particularly, the proposed method substantially reduced the processing time, to as low as 85 ms per image, compared to 140.3 and 182.6 ms per image in the two reference studies. Additionally, unlike mobile applications which place strict resource constraints, our application of smoky vehicle detection can work in a PC installed in a traffic surveillance office, which are not constrained by space, weight, and the impacts of mobility. In conclusion, the proposed method showed remarkable improvements in the accuracy, robustness, and feasibility of smoky diesel vehicle detection. Therefore, it offers potential to be applied in real-world situations.

Although the present study has demonstrated several improvements as mentioned above, compared to previous studies, we acknowledge some important limitations. First, the surveillance cameras are required to be set up at low positions. Second, we have not yet considered the dynamics of environmental conditions that may affect the model’s performance, such as occlusions, rapidly changing light, camera vibration, or sudden weather changes, which have been acknowledged in prior research [9]. Third, there are a number of adversarial attacks in real-world situations that might reduce the robustness of our model, such as the noise induced by the changing color of the road surface, bad weather conditions (like heavy rains), or partial occlusions by other vehicles or other objects on the roads. To address these issues in our future work, we will diversify the training data using two approaches: (1) collecting diverse data in various real-world scenarios, e.g., changing the color of the road surface, sudden weather changes (like sunshine to mist or sunshine to rain), different light intensities (from very bright to very dark in daytime and at night), partial occlusions over the smoke region by other vehicles or other objects on the roads; (2) using additional augmentation techniques that have yet to be included in the YOLO series, like blurring, adding noise, adding blocks to resemble partial occlusion, increasing and decreasing the brightness of figures, causing motion effects in images, etc.

Furthermore, we will use the results of smoky vehicle detection together with the identification of numbers on the license plate to exactly identify the smoky vehicles and automatically report to the environmental protection office to effectively manage smoky diesel vehicles. In addition, the proposed method in this study can be applied to detect the smoke region and use it as an input to a CNN to classify the level of gas emission from smoky vehicles.

## Figures and Tables

**Table 1:** Dataset for the prediction model of smoky diesel vehicles.

	Training Data	Valid Data
Images	5500	1315
Labels	20,200	4730
Ratio	80%	20%

**Table 2:** Computer configuration for the experiment problem.

Configuration	Parameter
CPU	AMD Ryzen 9 5950X 16-core processor 3.40 GHz
RAM	64.0 GB
GPU	NVIDIA GeForce RTX 3080 10 GB
YOLOv5	v6.0
Python	3.9.12
Pytorch	1.10.1
CUDA	11.3
cudnn	7.6.5

**Table 3:** Thirteen cases of matching for three classes (smoke, vehicle shape, and license plate) and corresponding outputs: (-: none; ✓: exist; ●: result).

Case	Detected Classes			Matching		Output	
	Truck	Plate	Smog	Smog∈Truck	Smog∈Plate	Smoky Vehicle	Non-Smoke Vehicle
1	-	-	-	-	-		●
2	✓	-	-	-	-		●
3	-	✓	-	-	-		●
4	-	-	✓	-	-		●
5	✓	✓	-	-	-		●
6	✓	-	✓	✓	-	●	
7	✓	-	✓	-	-		●
8	-	✓	✓	-	✓	●	
9	-	✓	✓	-	-		●
10	✓	✓	✓	-	-		●
11	✓	✓	✓	✓	✓	●	
12	✓	✓	✓	✓	-	●	
13	✓	✓	✓	-	✓	●	

**Table 4:** The performance of the four YOLOv5 models.

Model	Class	Precision	Recall	mAP@0.5	mAP@0.5:0.95
YOLOv5n	Truck	0.884	0.952	0.955	0.87
	Plate	0.885	0.918	0.94	0.568
	Smog	0.896	0.881	0.904	0.411
	All classes	0.888	0.917	0.933	0.616
YOLOv5s	Truck	0.893	0.951	0.961	0.881
	Plate	0.9	0.921	0.946	0.582
	Smog	0.914	0.883	0.91	0.421
	All classes	0.902	0.918	0.939	0.628
YOLOv5m	Truck	0.885	0.966	0.965	0.901
	Plate	0.896	0.936	0.949	0.593
	Smog	0.905	0.869	0.889	0.408
	All classes	0.895	0.923	0.934	0.634
YOLOv5l	Truck	0.883	0.971	0.962	0.897
	Plate	0.896	0.925	0.941	0.592
	Smog	0.903	0.87	0.908	0.412
	All classes	0.894	0.922	0.938	0.633

**Table 5:** Evaluation of proposed model with test data.

Methods	TP	FP	TN	FN	DR (%)	Precision (%)	FAR (%)	Processing Time (ms)
YOLOv5s + smoke–vehicle matching	95	23	124	23	80.51	80.51	15.65	53
YOLOv5s + smoke–license plate and Smoke–vehicle matchings	115	11	136	3	97.45	91.27	7.48	55
YOLOv5s + smoke–license plate and Smoke–vehicle matchings + smoke region refinement	115	8	139	3	97.45	93.50	5.44	85

**Table 6:** Comparison between our method and the state-of-the-art methods.

Methods	Test Data	DR (%)	Precision (%)	FAR (%)	Processing Time (ms)
Tao et al. [12]	Tao’s data	88.64	86.30	17.23	140.3
Peng et al. [17]	Peng’s data	77.13	79.36	1.92	182.6
Proposed method	Our data	97.45	93.50	5.44	85

## Data Availability

Data are contained within the article.
